# Targeted genome reconstruction strategy for endosymbionts in eukaryotic genomes

**DOI:** 10.1186/1753-6561-6-S6-P33

**Published:** 2012-10-01

**Authors:** Surya Saha, Wayne B Hunter, Magdalen Lindeberg

**Affiliations:** 1 Department of Plant Pathology and Plant-Microbe Biology, Cornell University, Ithaca, NY, USA; 2 USDA-ARS, US Horticultural Research Laboratory, Fort Peirce, FL, USA

## Background

The human microbiome project [1] is the first systematic and large-scale survey of microbial communities present in a eukaryotic metagenome. The availability of low-cost high-throughput next-generation sequencing technologies that enable us to sequence eukaryotes to a high depth of coverage presents an opportunity for *in silico* discovery of endosymbionts. We describe a method for mining a whole genome shotgun metagenome from an insect to identify members of the endosymbiont community, followed by reconstruction and validation of a high-quality draft microbial genome. The Asian citrus psyllid (*Diaphorina citri* Kuwayama or ACP) is host to 7+ bacterial endosymbionts [2] and is the insect vector of *Candidatus Liberibacter asiaticus* (Las), which is the causal agent of citrus greening, a disease costing the Florida citrus industry US$3.63 billion and 6,611 jobs since 2006. Citrus greening is a complex patho-system that involves interactions between the psyllid vector, the citrus host, and *Candidatus Liberibacter asiaticus*.

## Materials and methods

To gain a better understanding of the ACP endosymbiont community, DNA from ACP was sequenced to 108X coverage to produce paired-end and mate-pair libraries. Initial analyses focused on *Wolbachia*, an alpha-proteobacterial primary endosymbiont typically found in the reproductive tissues of ACP and other arthropods. The metagenomic sequences were mined for *Wolbachia* (wACP) reads using four sequenced *Wolbachia* genomes as bait. Putative wACP reads were then assembled using Velvet and MIRA3 assemblers. The resulting wACP contigs were annotated using the RAST pipeline and compared with the closest sequenced *Wolbachia* from an insect genome: *Wolbachia* endosymbiont of *Culex quinquefasciatus* (wPip). MIRA3 was able to reconstruct a majority of the wPip coding sequence regions and was therefore selected for scaffolding using large insert mate-pair libraries. The wACP scaffolds were further improved using Abacas and Mauve contig mover with wPip as reference genome to orient and order the contigs.

## Results and conclusions

We validated the final wACP scaffold by comparing all wACP proteins against the four sequenced *Wolbachia* genomes. We ran OrthoMCL and selected core and shared *Wolbachia* proteins where the classification was highly conserved (>80%) across all runs. In order to determine the presence of the core *Wolbachia* proteins in our wACP scaffold, we compared wACP proteins with labeled *Wolbachia* proteins. 1,164/1,213 wACP proteins had matches, of which 669 were to core proteins. This number compares favorably to the number of core proteins (670) found in sequenced *Wolbachias.* The scope of endosymbiont characterization was expanded beyond wACP using 16S rDNA and partial 23S rDNA analysis [2] as a guide. Reads from the ACP metagenome were mapped to reference genomes of candidate endosymbionts and mapped regions were analyzed manually. We are currently screening out rRNA regions and using taxonomic classification in order to determine the specificity of these regions to the genome. Results will be presented regarding targeted assembly methods, validation metrics and effectiveness of taxonomic classification strategies for metagenomics.
